# Supplementary material for: Conjunctive Analyses of BSA-Seq and BSR-Seq Unveil the Msβ-GAL and MsJMT as Key Candidate Genes for Cytoplasmic Male Sterility in Alfalfa (Medicago sativa L.)
Source: Int J Mol Sci. 2022 Jun 28;23(13):7172. doi: 10.3390/ijms23137172 (PMC9266382; doi:10.3390/ijms23137172)
Supplement: Supplementary file 1 [file ijms-23-07172-s001.zip › ijms-1783602-supplementary.pdf]

Supplementary materials

Tables

Table S1. The cytoplasmic male sterile line fertility classification standard of alfalfa in this study.

Fertility classification	Pollen abortion rate (%)
Fertility	95.1-100
Sterility	0-10

Table S2. The specific primer sequence used in qRT-PCR vaklidation

No.	Gene	Gene_id	Primer sequence (5'-3')
1		c115887.graph_c0-F	TGGCTCTCACAGGTTCCTAAA
		c115887.graph_c0-R	TCATGGTAAGCGTTGAGCAC
2		c101056.graph_c0-F	TCACGCTGATGGTGCTCT
		c101056.graph_c0-R	TACCCTGCCTTGTGTTGC
3		c102778.graph_c0-F	AGTTTGGGCTGGATTTTT
		c102778.graph_c0-R	TTGTTTTAGGCTCGTTGG
4		c101072.graph_c0-F	CATTTCTGCATCTTTTCG
		c101072.graph_c0-R	CATTCTGGACCTCTATCG
5		c102786.graph_c0-F	TAAGGTAAGTGTGGAAGA
		c102786.graph_c0-R	AAGTTTGAGATTATGGTG
6		c111356.graph_c0-F	ACGACGACGAAGACGGAGACC
		c111356.graph_c0-R	TGGAGGGAGGAACCCACATGG
7		c104110.graph_c0-F	GATGTTGACACCCTATCC
		c104110.graph_c0 -R	ATGAGTTTCACCCTCTTT
8		c120364.graph_c1-F	TTGTTGAGAAGGGTGACA
		c120364.graph_c1-R	AAGATAATCGGAGAGGAG
9		c102760.graph_c0-F	ATTCTGGGGCTGTTGATA
		c102760.graph_c0-R	CTGACCCTCCTTCCTTTT
00	Reference	Actin -F	CTGGAATTGCTGACCGTATGAG
		-R	ATGGATCCTCCAATC

Table S3. Separation ratios of sterile and fertile plants.

Sample	Extremely Sterile Plants (T03)	Extremely fertile plants (T04)	Expected ratio	χ^2	ρ
F2 segregation population	16	272	1:15	0.237	0.626

Figures

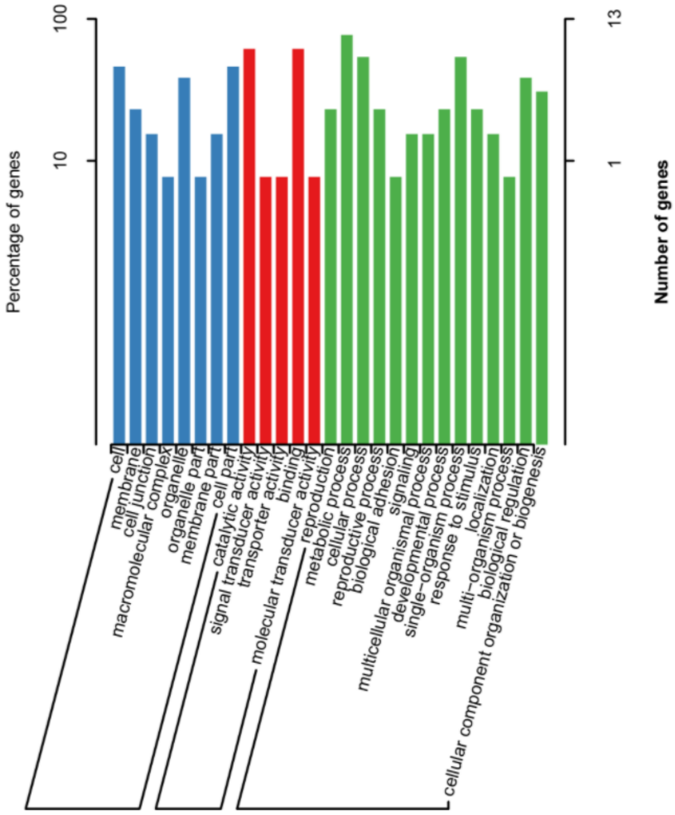

Figure S1. The GO annotation of genes in candidate region.
